# Incorporating pharmacodynamic considerations into caffeine therapeutic drug monitoring in preterm neonates

**DOI:** 10.1186/s40360-016-0065-x

**Published:** 2016-06-07

**Authors:** Tian Yu, Alfred H. Balch, Robert M. Ward, E. Kent Korgenski, Catherine M. T. Sherwin

**Affiliations:** Division of Clinical Pharmacology, School of Medicine, University of Utah, 295 Chipeta Way, Suite 1C310, Salt Lake City, UT 84108 USA; Division of Neonatology, Department of Pediatrics, School of Medicine, University of Utah, Salt Lake City, Utah 84108 USA; Intermountain Healthcare, Salt Lake City, Utah 84108 USA

**Keywords:** Caffeine, Neonate, Pharmacodynamics, Therapeutic drug monitoring

## Abstract

**Background:**

This study sought to assess the pharmacokinetic and pharmacodynamic relationships of caffeine citrate therapy in preterm neonates who had therapeutic drug monitoring (TDM) in the post-extubation period.

**Methods:**

A retrospective observational study was conducted in preterm neonates who received caffeine citrate therapy for apnea of prematurity and had TDM done in the post-extubation period between January 2006 and October 2011. The relationships between pharmacodynamic effects (heart rate, respiratory rate, episodes of apnea, adverse events) and caffeine serum concentrations were explored.

**Results:**

A total of 177 blood samples were obtained from 115 preterm neonates with a median (range) gestational age of 29 (24 – 33) weeks and birth weight of 1230 (607 – 2304) kg. Caffeine citrate therapy was initiated at a median (interquartile range) postnatal age of 1 (1 – 3) day and TDM was performed at a postnatal age of 15 (10 – 24) days. No direct correlations were found between respiratory rate or apneic episodes and caffeine serum concentrations; however, heart rate and caffeine serum concentrations were significantly correlated (*p* < 0.05). Dosing regimen of 40/5 mg/kg q12h (loading dose/maintenance dose, time interval) led to similar endotracheal re-intubation rate but increased percentage of patients experiencing tachycardia compared to the standard regimen of 20/5 mg/kg q24h (44.7 % vs 10.2 %, *p* < 0.001).

**Conclusion:**

Based on this retrospective study, no correlation between episodes of apnea and caffeine serum concentrations was found in neonates who had TDM of caffeine citrate therapy in the post-extubation period, whereas a significant association between tachycardia and concentrations existed. Notwithstanding the absence of severe adverse reactions, TDM should be considered in critically ill neonates with unexplained adverse effects, such as tachycardia.

**Electronic supplementary material:**

The online version of this article (doi:10.1186/s40360-016-0065-x) contains supplementary material, which is available to authorized users.

## Background

Caffeine citrate is the first-line therapy for treatment of apnea of prematurity [1, 2]. The standard dosing regimen, approved by FDA in 1999 [3], is an intravenous loading dose of 20 mg/kg caffeine citrate followed by a maintenance dose of 5 mg/kg daily. Other dosing regimens exist, contingent upon the neonatologist’s decision based on the neonate’s disease status. The therapeutic range of 5 – 20 mg/L has been used to guide dosing in neonates and has its origin in early works by J. V. Aranda et al., which reported that optimizing ventilatory drive and control of apnea without toxicity were achievable with caffeine serum concentrations within this range [4]. Clinical signs of toxicity have been observed with caffeine serum concentrations above 40 mg/L [5]. However, therapeutic drug monitoring (TDM) is not routinely performed due to its benign safety profile when standard dosing is used [6, 7]. Since TDM usually requires blood sampling by heel prick and can contribute to anemia, it is important to understand if TDM is necessary during caffeine citrate therapy, especially with varied dosing regimens other than the FDA approved regimen.

Several studies did not support the practice of routine TDM of caffeine citrate therapy or indicated higher upper bound of therapeutic range (>20 mg/L) in preterm neonates [8–10]. An observational study of 101 preterm neonates revealed that standard maintenance dose led to serum concentrations within the recommended range (5 – 20 mg/L) independent of gestational age (GA), which also held true for patients with concomitant renal or hepatic dysfunction [8]. A prospective study reported that caffeine serum concentrations from standard dosing were in a safe and therapeutic range of 11 – 33 mg/L by 14 postnatal days and were independent of neonatal demographics including GA, postmenstrual age (PMA), and weight, suggesting that routine TDM was not necessary without clinical signs of apnea or toxicity [9]. A population pharmacokinetic (PK) study showed that the inter-occasion (day-to-day) variability of caffeine clearance was twice the inter-individual variability in preterm neonates, which implied that adjusting a maintenance dose based on previous serum concentrations of the individual neonate was not effective due to the marked day-to-day randomness in clearance [10]. Gal suggested that the therapeutic concentrations could range from 10 to 40 mg/L and the likelihood of response and toxicity were specific to each individual [11, 12].

Our study examined cardiovascular/respiratory effects including heart rate, respiratory rate, episodes of apnea, and adverse events as major pharmacodynamic (PD) parameters and delineated their association with caffeine serum concentrations in preterm neonates. Clinical interventions and adverse events were also compared among various dosing regimens to illustrate the aggregated clinical outcomes resulting from different caffeine exposures. The aim of this study was to find the relationship between PD responses and caffeine serum concentrations to inform the use of TDM in neonates. This retrospective study was conducted to serve as a preliminary work for a future prospective study on caffeine citrate dosing regimen optimization in preterm neonates.

## Methods

### Study design

This retrospective observational study consisted of preterm neonates who received caffeine citrate for apnea of prematurity at 8 sites of Intermountain Healthcare System in Utah (Utah Valley Hospital, Intermountain Medical Center, McKay-Dee Hospital, Primary Children’s Hospital, Dixie Regional Hospital, Latter-day Saints Hospital, American Fork Hospital, Logan Regional Hospital) between January 2006 and October 2011. Neonates who had been previously ventilated at birth, received at least 1 dose of intravenous caffeine citrate within 28 days of postnatal age (PNA), and had at least 1 blood sample taken for caffeine concentration measurement were included for analysis. Patients were excluded if they received caffeine citrate therapy for other indications (neonatal respiratory distress syndrome), apnea due to other causes (confirmed sepsis or pneumonia, diagnosed gastroesophageal reflux), or had no TDM. Preterm neonates with abnormalities in central nervous system were also excluded as the disease may affect the patient response to caffeine. Patient demographics including gender, birth weight, APGAR 1 min score, APGAR 5 min score, GA, PNA at dosing or sampling, PMA at dosing or sampling were recorded in enterprise data warehouse. This study was reviewed, approved, and granted a waiver of informed consent by the University of Utah Institutional Review Board.

### Sample collection and measurement

The information on doses, dosing intervals, and TDM sample concentrations were obtained from enterprise data warehouse of Intermountain Healthcare System. In the clinical settings, a loading dose of either 40 mg/kg (20 mg/kg caffeine base equivalent) or 20 mg/kg caffeine citrate (10 mg/kg caffeine base equivalent) was administered by intravenous infusion over 15 min, followed by a maintenance dose of 5 mg/kg caffeine citrate (2.5 mg/kg caffeine base equivalent) every 12 or 24 h through intravenous or orogastric/nasogastric routes. The dosing regimen is denoted as loading dose/maintenance dose, dosing time interval throughout this paper. Decisions regarding caffeine citrate dosing regimen, use of TDM, and timing of blood sample acquisition were made by the clinical staff. The indication for TDM was inadequate responses such as repeated or severe apnea despite the continuation of caffeine therapy or symptoms of adverse events [13, 14]. The timing of sample collection post last dose was random and was at the discretion of the care providers. Total caffeine concentrations in serum were measured by quantitative enzyme multiplied immunoassay (EMIT caffeine assays, Siemens Healthcare, Pennsylvania, USA). The assay was accurate between 1 and 30 mg/L with between-day and within-day imprecision < 10 % across this range [15]. Samples with caffeine concentration > 30 mg/L were diluted in drug-free serum and reanalyzed.

### Clinical records identification and analysis

Intermountain HELP2 Clinical Desktop was searched to obtain clinical notes concurrent with sample collections using the patient’s enterprise master patient index number. The clinical notes included relevant critical care progress notes or discharge summaries. Vital signs (heart rate and respiratory rate) were recorded in the critical care progress notes on the date of sample collection along with the frequency of apnea and any adverse effects attributed to caffeine. The normal range of heart rate in neonates is 120 – 160 beats per minute (b · min^−1^) with toxicity associated with > 220 b · min^−1^. Episodes of heart rate > 170 b · min^−1^ were considered as tachycardia according to the clinical notes. The respiratory rate normal range in neonates is 30 – 60 breaths per minute (br · min^−1^) with toxicity defined as tachypnea > 80 br · min^−1^. Information on apnea or adverse events was screened in the notes. Apnea was cessation of breathing lasting > 20 seconds, and/or those that were shorter, but associated with hypoxia (oxygen saturation < 85 %) or bradycardia (heart rate < 100 b · min^−1^). Episodes of apnea (number) or onset of adverse events (yes/no) that happened on the same day of sample collection were recorded. If critical care progress notes concurrent with sample collections were not available, the apnea or adverse event records from the hospital discharge summary were searched to identify any information on apneic episodes or adverse events on the date of sample collection. Records from patients who were not on mechanical ventilators on the days of TDM were used to evaluate the relationships between vital signs/apnea episodes and caffeine serum concentrations. It is to note that vital signs were measured during the physical exam on the same day of TDM as dictated by the attending clinician, thus may not reflect the heart rate in a tachycardia event or respiratory rate in an apneic episode.

Clinical intervention in terms of endotracheal re-intubation was used as a surrogate marker of treatment failure in this study. Patients requiring endotracheal re-intubation during the entire course of caffeine citrate therapy were identified in the clinical notes. The underlying indications for re-intubation were divided into two subgroups, one subgroup was re-intubation secondary to worsening symptoms of apnea of prematurity, the other subgroup was re-intubation secondary to other respiratory failure etiologies, such as significant periodic breathing, increased work of breathing, increased CO_2_ levels, significant episodes of bradycardia with desaturations, pleural effusions, pulmonary edema, and suspected infections (no culture confirmation).

### Statistics

Differences in caffeine serum concentrations between dosing regimen groups were determined by Mann–Whitney *U* test. Linear regression was used to evaluate the association between PD effects and caffeine serum concentrations. The relationships between treatment efficacy and patient demographics were also evaluated by linear regression. Predicted probabilities for adverse events as a function of caffeine serum concentrations were assessed by logistic regression analysis. The number of patients requiring re-intubation or experiencing adverse events was compared among various dosing regimens via a *χ*2 test of independence. Statistics were performed using SAS software (version 9.3) (SAS Inc. North Carolina, USA) and differences were considered significant at *p <* 0.05.

## Results

A total of 115 preterm neonates who received caffeine citrate therapy and underwent TDM in the post-extubation period were included in this study. A total of 177 blood samples were taken from these patients with a median (interquartile range) GA of 29 (28 – 30) weeks and birth weight of 1230 (997 – 1485) g (Table 1). Caffeine citrate therapy was started in patients at PNA of 1 (1 – 3) day or PMA of 29.4 (28.1 – 30.7) weeks. Blood samples were taken in patients with PNA of 15 (10 – 24) days or PMA of 31.6 (30.1 – 33.4) weeks (Table 1). Patients had a median of one sample taken for TDM. As shown in Table 2, 47 (40.9 %) patients received 40/5 mg/kg q12h regimen, 49 (42.6 %) patients received 20/5 mg/kg q24h. The dosing regimen of 40/5 mg/kg q12h led to significantly higher concentrations compared with the standard regimen 20/5 mg/kg q24h (median 23 vs 15 mg/L, *p* < 0.001). Patient demographics and clinical statuses were similar among dosing regimen groups (data not shown).

**Table 1:** Demographics of preterm neonates included in this study

Characteristics	Median (Interquartile range)	Range
Sex^a^	60 male, 55 female	
GA (week)	29 (28 – 30)	24–33
Birth weight (g)	1230 (997–1485)	607–2304
Apgar score, 1 min	6 (4–8)	1–9
Apgar score, 5 min	8 (7–9)	3–9
PNA at initiation (day)	1 (1–3)	0–25
PMA at initiation (week)	29.4 (28.1–30.7)	24.1–33.6
PNA at sampling (day)	15 (10–24)	3–84
PMA at sampling (week)	31.6 (30.1–33.4)	25.6–40.9

^a^Number

**Table 2:** Caffeine citrate dosing regimens and caffeine serum concentrations during TDM

Dosing regimens	Number of patients	Caffeine serum concentration (mg/L)
40/5 mg/kg		
q12h	47	23 (18–26)^***^
20/5 mg/kg		
q24h	49	15 (11–17)
q12h	8	17 (13–20)
5/5 mg/kg		
q24h	6	13 (8–17)
q12h	5	22 (21–23)

Data = median (interquartile range)

^***^ *p* < 0.001, significant differences were observed in caffeine serum concentrations between regimen 40/5 mg/kg q12h and standard 20/5 mg/kg q24h

**Fig. 1:**
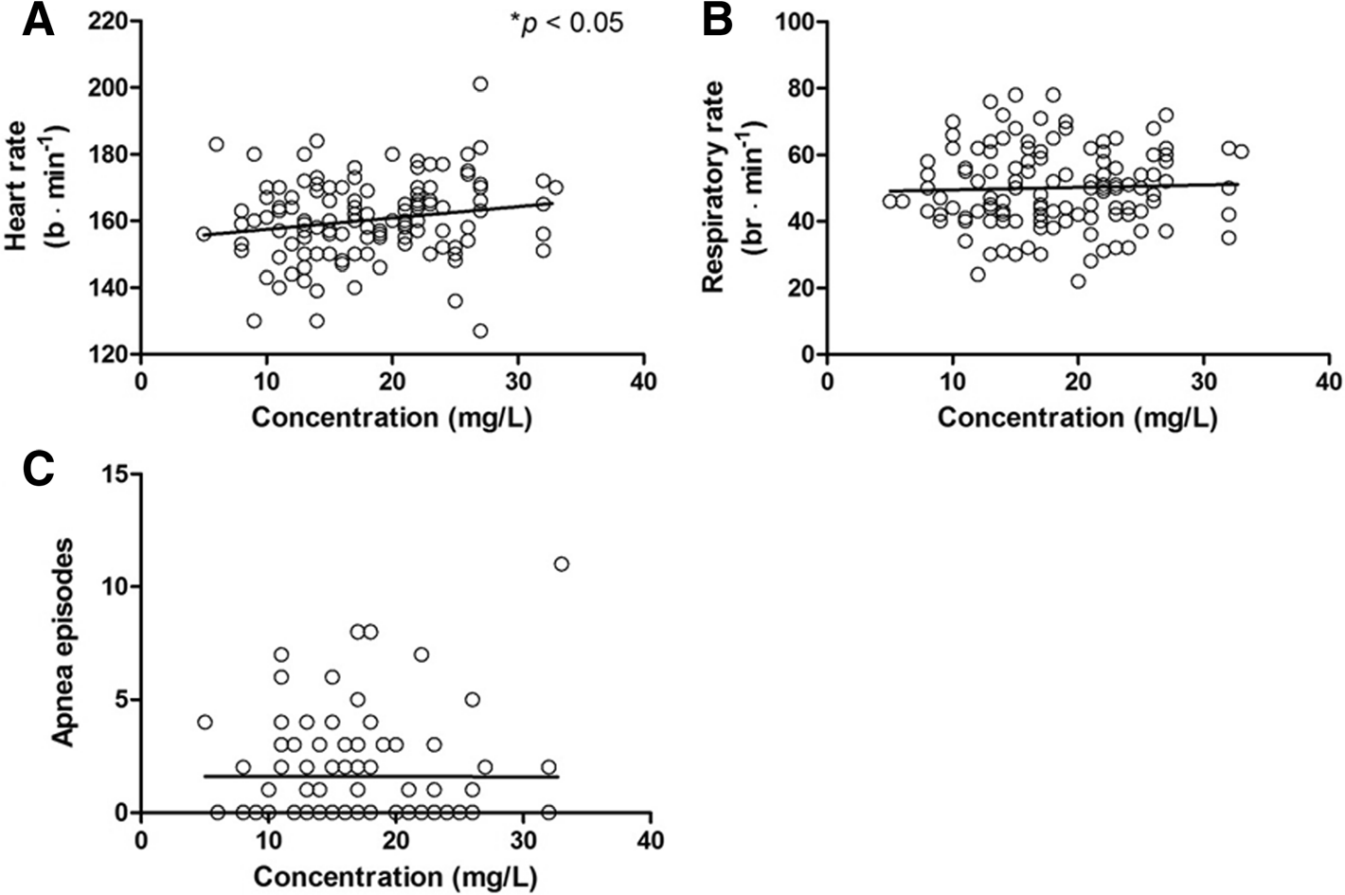
Relation of **a** heart rate (r^2^ = 0.031, *p* = 0.04), **b** respiratory rate (r^2^ = 0.001, *p* = 0.68), **c** episodes of apnea (r^2^ < 0.001, *p* = 0.97) recorded during the physical exam in the day of TDM as a function of caffeine serum concentration. Note: b · min^−1^, beats per minute; br · min^−1^, breaths per minute

**Fig. 2:**
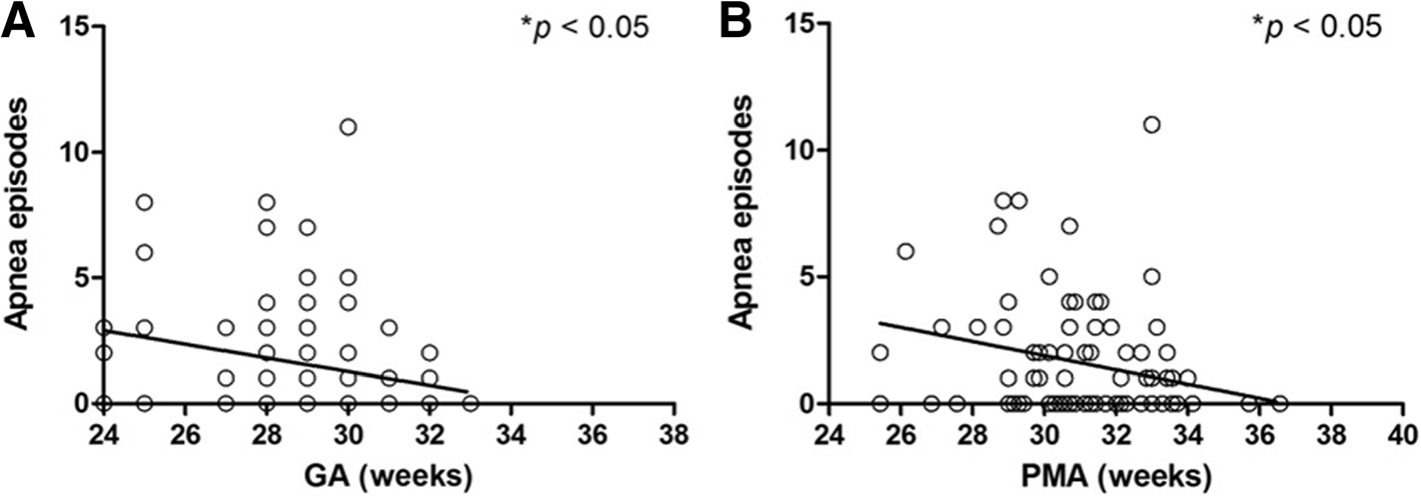
Number of apnea episodes in the day of TDM as a function of **a** GA (r^2^ = 0.061, *p* = 0.01), **b** PMA (r^2^ = 0.071, *p* = 0.01) in preterm neonates

Out of 177 concentrations collected, 149 (84.2 %) concentrations had concurrent clinical notes that provided relevant PD information. Among the 149 concentrations, 125 (83.8 %) concentrations had concurrent heart rate and respiratory rate available in non-ventilated neonates, whereas 89 (59.7 %) concentrations had corresponding definitive number of apneic episodes recorded in non-ventilated neonates. Linear regression analysis on heart rate, respiratory rate, and episodes of apnea as a function of caffeine serum concentrations showed that heart rate was significantly associated with concentration (*p* < 0.05) (Fig. 1a). Although this relationship was statistically significant, the physiological effect was considered small potentially due to the fact that retrospective records were used. The median (range) of heart rates at caffeine serum concentration of 5–10 mg/L and 30–35 mg/L were 160 (130–183) and 168 (151–175) b · min^−1^, respectively. No linear relationships were found for the other PD parameters (Fig. 1b, c). The relations between apnea episodes in the day and patient demographics were assessed. It was found that there was an inverse relationship between number of apnea episodes in the day and GA of neonates (*p* < 0.05) (Fig. 2a). Frequency of apnea episodes decreased significantly as PMA increased (*p* < 0.05) (Fig. 2b).

**Fig. 3:**
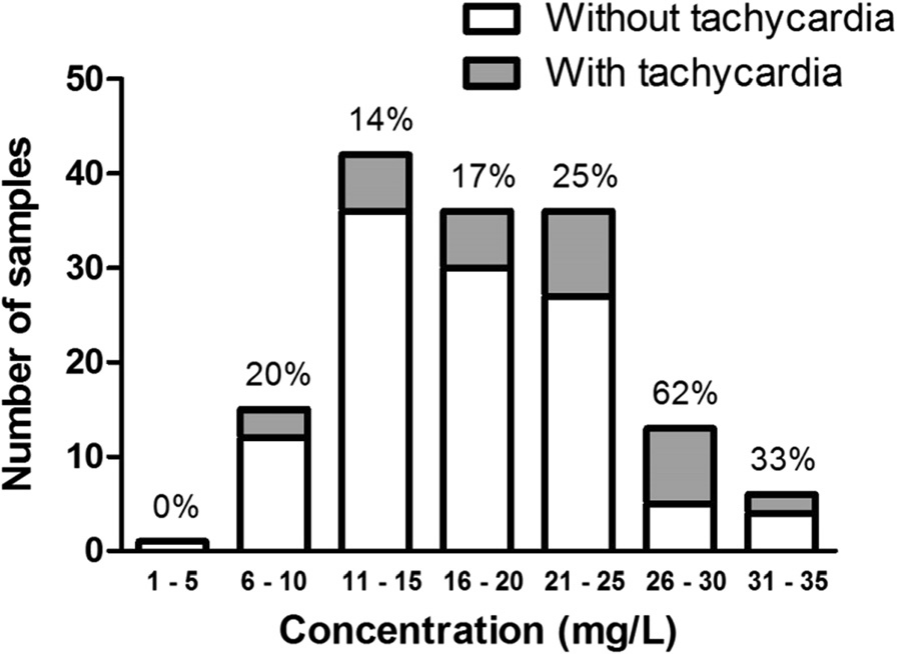
The frequency of tachycardia or the percentage of samples with tachycardia relative to caffeine serum concentration in preterm neonates

**Fig. 4:**
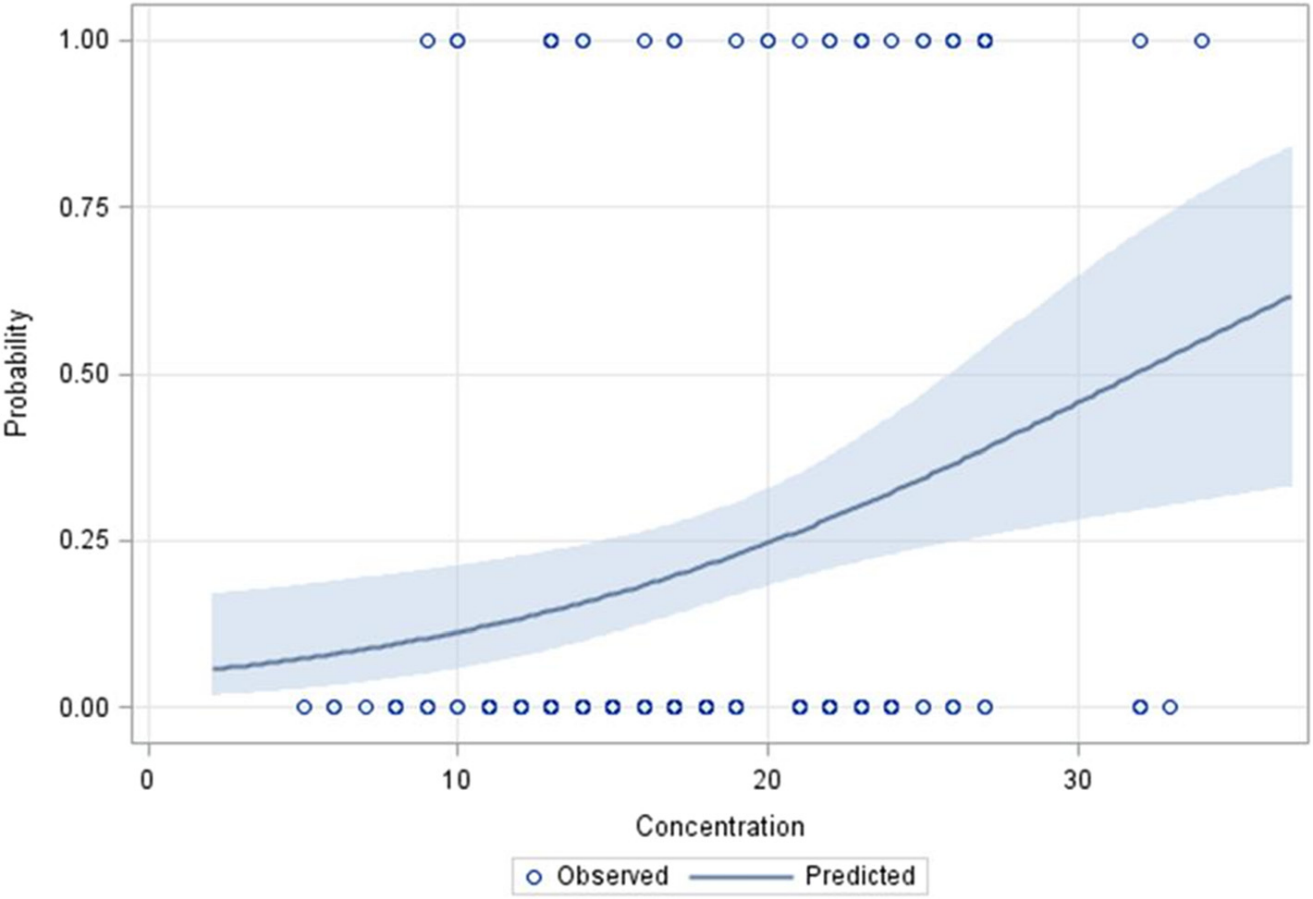
Predicted probabilities of tachycardia as a function of caffeine serum concentration in preterm neonates. An open circle represents the presence (probability = 1) or absence (probability = 0) of a tachycardia event at the corresponding concentration. The solid line with blue band represents estimated probability and the 95 % confidence limits

The most common adverse event during caffeine TDM was tachycardia. Out of 149 concentrations with concurrent medical records documented, there were 34 (22.8 %) concentrations associated with tachycardia, 3 (2.0 %) with tachypnea, and 1 (0.7 %) with mild hypertension, which were considered to be secondary to caffeine citrate therapy. The severity of recorded tachycardia was mostly mild to moderate with heart rate ranging from 170 – 212 b · min^−1^ amid an episode. The frequency of tachycardia or the percentage of samples with tachycardia relative to the concentration was plotted in Fig. 3. Predicted probabilities for tachycardia events as a function of caffeine serum concentrations were also shown in Fig. 4.

Out of 115 patients, a total of 27 patients (23.4 %) were re-intubated onto a mechanical ventilator during the entire course of caffeine citrate therapy, of which 10 patients (8.6 %) were re-intubated secondary to worsening symptoms of apnea. As shown in Table 3, there was no difference in re-intubation rate secondary to apnea of prematurity or other pulmonary etiologies between regimen 40/5 mg/kg q12h and standard 20/5 mg/kg q24h, however, regimen 40/5 mg/kg q12h led to significantly higher percentage of patients experiencing tachycardia than the standard regimen (*p* < 0.001). Patients going through re-intubation tend to be more preterm [mean (range) GA 27 (24–31) weeks] with less birth weight [963 (607 – 1892) g] than average statistics of this study population (Table 1). There was no difference in these patients’ demographics among dosing regimens (Additional file 1: Table S1).

**Table 3:** Summary of clinical interventions/adverse events in terms of re-intubation and tachycardia among dosing regimens

Dosing regimens	Number of patients going through re-intubation (%)		Number of patients having tachycardia (%)
	Secondary to apnea of prematurity	Secondary to other respiratory etiologies	
40/5 mg/kg			
q12h	4 (8.5 %)	7 (14.9 %)	21^***^ (44.7 %)
20/5 mg/kg			
q24h	4 (8.2 %)	8 (16.3 %)	5 (10.2 %)
q12h	0	1 (12.5 %)	4 (50.0 %)
5/5 mg/kg			
q24h	2 (33.3 %)	0	1 (16.7 %)
q12h	0	1 (20.0 %)	0

^***^ *p* < 0.001, regimen 40/5 mg/kg q12h led to significantly higher percentage of patients experiencing tachycardia secondary to caffeine citrate therapy than standard 20/5 mg/kg q24h

## Discussion

The present study revealed that a correlation between number of apneic episodes and caffeine serum concentrations was not established under current dosing regimens. A significant association between heart rate and concentrations was found among the other PD parameters, consistent with the fact that the probability of tachycardia increased as caffeine serum concentration increased. A high dose regimen 40/5 mg/kg q12h led to similar re-intubation rate but significantly higher percentage of patients having tachycardia than standard regimen 20/5 mg/kg q24h, agreeing well with the PK/PD relationships found above. The total re-intubation rate of the standard regimen in our patients (24.5 %) was similar to that reported in the literature (24.0 %) [16].

The lack of correlation between efficacy and caffeine serum concentrations under current dosing regimens was in agreement with the trial that granted caffeine citrate label approval by FDA, which reported no association between success of ≥ 50 % reduction or elimination in apnea events and mean daily caffeine concentrations.^3^ Skouroliakou et al.’s study also revealed that methylxanthine concentrations were not significantly associated with number of apneic events per day in neonates with GA < 33 weeks [17]. A previous study comparing 10 mg/kg and 5 mg/kg maintenance doses in neonates with GA < 32 weeks showed similar efficacy in reducing apnea spells despite significantly higher frequency of tachycardia in the 10 mg/kg group [18]. It could be partially due to the underlying multifactorial etiology of apnea, with prematurity being a prerequisite for the indication [19]. Apnea of prematurity is known to have an incidence inversely related to GA and could regress with the maturation of the newborn [19]. This is echoed in our results that the number of apnea episodes reduced significantly as PMA increased as well as in patients with higher GA (Fig. 2).

The variable PK/PD response in neonates to caffeine citrate therapy is also likely to be attributed to variability in caffeine metabolism in individuals. Caffeine metabolism by hepatic enzymes is usually limited in neonates. Maturation of metabolic enzymes could lead to the improvement in metabolic function, which is significantly associated with the increase in PNA and varies extensively among individuals (range 1 %–41 %) [20]. N7-demethylation, which produces theophylline, acts as the predominant metabolic pathway in premature neonates (range 1 %–37 %) [20]. Theophylline is the active metabolite that is partially responsible for side effects such as tachycardia [18], the variable caffeine-theophylline conversion rate could lead to PD response variability. Several other factors, including genetic variation in hepatic metabolic enzymes and genetic variations in caffeine receptors may also contribute to the variability in PD responses [21–23]. Due to the variability in the caffeine metabolism and the resulting wide range of half-lives among individuals [10], the measurement of caffeine serum concentrations at a postnatal age of 15 (10–24) days in our study population may not necessarily reflect concentrations at steady state. Thus, caffeine concentrations may vary considerably and make it difficult to find other significant associations between PD responses and concentrations.

The 2-fold higher-than-standard dosing regimen led to similar re-intubation rate but significantly higher percentage of patients having tachycardia than standard regimen. This is similar to Steer et al.’s findings on the use of 3–6 fold higher maintenance doses for a course of 7 days in neonates of similar GA range (<32 weeks) to our study’s (<33 weeks) [24]. A significant reduction in re-ventilation was shown when 4-fold higher maintenance doses were used for the duration of averagely 1 month in neonates with GA < 30 weeks [16]. This effect was more evident in the stratified subgroup of neonates with GA < 28 weeks that a significant reduction in the days on mechanical ventilator (average 8 days) was observed [16]. A retrospective study in neonates < 28 weeks GA revealed that patients receiving caffeine citrate > 7.9 mg/kg/day were associated with a decreased need for clinical interventions in terms of dose adjustments compared to those receiving ≤ 7.9 mg/kg/day doses [25]. It suggests that neonates with lower GA, especially extremely low-gestational-age neonates (GA < 28 weeks), could benefit more from high doses of caffeine citrate. Dosing regimens stratified by GA is warranted for more systematic trial evaluation.

None of the neonates died or had a severe reaction under current dosing regimens, however, TDM may be helpful in suspected toxicity to diagnose caffeine-related adverse events, based on our findings on a significant association between tachycardia and caffeine serum concentrations. The use of high dose caffeine citrate inevitably increases the risk of tachycardia in neonates, this need to be taken into consideration combined with other elements of therapy, such as efficacy and requirement for respiratory support, to determine treatment priority in lieu of medical cost and facility resources available. Caffeine TDM is valuable in the setting of clinically-significant tachycardia to assist differential diagnosis with respect to other potential etiologies.

This study has several limitations associated with its nature of retrospective chart evaluation. First, the TDM was done at the discretion of the medical team and may have patient selection bias towards sicker patients or patients who had adverse events. Second, vital signs were measured during the physical exam in the day of TDM, thus, they were the approximate rather than the exact values at TDM sampling time. Third, the records were physician notes that were not verified by audits of electronic monitoring, however, they are the critical information used by clinicians for patient management and decision making.

## Conclusion

Based on our analysis on the retrospective dataset, little correlation between episodes of apnea and caffeine serum concentrations was observed in neonates who had TDM in the post-extubation period under current dosing regimens, whereas a significant association between tachycardia and concentrations existed. Notwithstanding the absence of severe adverse reactions, TDM should be considered in critically ill neonates with unexplained adverse effects, such as tachycardia. Future prospective study is warranted to establish the linked PK/PD relationship to optimize dosing regimen in preterm neonates.

### Availability of supporting data

Supporting data are available in the form of an extended study report to the ethics committee of the University of Utah Institutional Review Board. This report is available upon request, which should be addressed to the corresponding author.

## Additional file

Additional file 1: Table S1 Demographics of preterm neonates stratified by major dosing groups (DOCX 14 kb)

## Abbreviations

GA: gestational age
PD: pharmacodynamics
PK: pharmacokinetics
PMA: post-menstrual age
PNA: post-natal age
TDM: therapeutic drug monitoring
