# Supplementary material for: Incorporating pharmacodynamic considerations into caffeine therapeutic drug monitoring in preterm neonates
Source: BMC Pharmacol Toxicol. 2016 Jun 7;17:22. doi: 10.1186/s40360-016-0065-x (PMC4896039; doi:10.1186/s40360-016-0065-x)
Supplement: Additional file 1: — Table S1 Demographics of preterm neonates stratified by major dosing groups (DOCX 14 kb) [file 40360_2016_65_MOESM1_ESM.docx]

**Additional file 1:** Table S1 Demographics of preterm neonates stratified by major dosing groups

|  | **40/5 mg/kg q12h** | | **20/5 mg/kg q24h** | |
| --- | --- | --- | --- | --- |
| **Characteristics** | **Median (Interquartile range)** | **Range** | **Median (Interquartile range)** | **Range** |
| Gender^*^ | 21 male, 26 female | | 28 male, 21 female | |
| Gestational age (week) | 29 (28 – 30) | 24 - 32 | 29 (27 – 30) | 24 - 33 |
| Birth weight (g) | 1170 (986 – 1413) | 607 - 2304 | 1313 (1073 – 1535) | 665 - 2269 |
| Apgar score, 1 min | 6 (4 – 7) | 1 - 9 | 7 (5 – 8) | 1 - 9 |
| Apgar score, 5 min | 8 (7 – 9) | 4 - 9 | 8 (7 – 9) | 4 - 9 |
| PNA at sampling (day) | 11 (9 – 14) | 5 - 27 | 12 (8 – 17) | 3 - 27 |
| PMA at sampling (week) | 30.4 (29.4 – 31.9) | 25.4 – 35.6 | 30.9 (29.1 – 32.1) | 25.4 – 34.1 |

^*^Number.
